# Coordination Between Treg Cells and *Bifidobacterium* in the Immune–Bacterial Network of Human Colostrum

**DOI:** 10.1155/jimr/8677501

**Published:** 2026-06-22

**Authors:** Mextli Y. Bermejo-Haro, Graciela Hernández-Peláez, Ivonne J. Álvarez-Peña, Alma Herrera-Salazar, Diana Sarahi De la Merced-García, Anayansi Molina-Hernández, Carlos Domínguez-Vanegas, Jonatan A. Mendoza-Ortega, Rodrigo T. Camacho-Pacheco, M. Angel Najera-Hernandez, M. Fernanda Aguilar-Dueñas, Libier Cabrera-Rivera, Edna Basilio-Gálvez, Diana Soriano-Becerril, Noemí Plazola-Camacho, Ricardo Figueroa-Damián, Sandra Rodríguez-Martínez, Ismael Mancilla-Herrera

**Affiliations:** ^1^ Infectology and Immunology Department, National Institute of Perinatology, Mexico City, Mexico, inper.edu.mx; ^2^ Department of Immunology, National School of Biological Sciences, National Polytechnic Institute, Mexico City, Mexico, ipn.mx; ^3^ Graduate Program in Immunology, National School of Biological Sciences, National Polytechnic Institute, Mexico City, Mexico, ipn.mx; ^4^ Nursing Department, National Institute of Perinatology, Mexico City, Mexico, inper.edu.mx; ^5^ Physiology and Cellular Development Department, National Institute of Perinatology, Mexico City, Mexico, inper.edu.mx; ^6^ Immunobiochemistry Department, National Institute of Perinatology, Mexico City, Mexico, inper.edu.mx

**Keywords:** colostrum, delivery mode, gestational age, immune tolerance, Treg cells

## Abstract

Breast milk is the primary source of nutrients, bacterial, and defensive elements, which are required for infants in their first years of life. Colostrum, the first stage of breast milk, contains abundant levels of antibodies, lymphocytes, and commensal bacteria. The affinity, phenotype, and diversity of these components resemble those found in maternal enteric mucosa, suggesting that the enteromammary pathway facilitates their transport. In the gut, commensal bacteria, IgA, and regulatory T cells (Treg) are interrelated in maintaining immune tolerance and defense, leading us to hypothesize that similar correlations may exist in colostrum. In this study, we present a descriptive analysis of 33 colostrum samples collected from healthy women. DNA from *Staphylococcus*, *Streptococcus*, *Bifidobacterium*, *Lactobacillus*, and *Enterococcus* was quantified by quantitative PCR (qPCR). Immunoglobulin isotypes and cytokines were measured using multiplex immunoassays, and the Treg cell frequencies were determined by flow cytometry. Correlation tests and multivariate analysis were used to evaluate these associations. The results showed that *Streptococcus* and *Staphylococcus*, common bacteria found on the skin and predominantly in breast milk, were not significantly associated with immunoglobulins or Treg cells. Interestingly, *Bifidobacterium*, but not *Lactobacillus* or *Enterococcus*, showed a positive correlation with Treg cells. Contrary to our initial hypothesis, neither Treg cells or *Bifidobacterium* were negatively correlated with antibodies. These findings suggest a potential association between Treg cells and specific commensal bacteria, particularly *Bifidobacterium*, that appears to be independent of immunoglobulins. This cellular microbial interaction could be involved in neonatal gut colonization, immune tolerance, and early immune responses to antigenic challenges.

## 1. Introduction

Breast milk is the optimal source of bioactive factors that promote healthy development during the first year of life, with evidence supporting continued benefits into later stages of life [1]. The highly dynamic composition of breast milk adapts to fulfill the nutritional, developmental, microbial colonization, and immune defense requirements of breastfed infants [2].

Colostrum, defined as the milk secreted during the first 5 days postpartum, is a unique stage of lactation due to its exceptionally high content of immunological components compared to transitional milk (produced up to 2 weeks postpartum) and mature milk (produced during the remaining period of lactation) [3, 4]. Colostrum contains cytokines, chemokines, and growth factors that help mature mucosal barriers and improve the immune system of breastfed newborns. Additionally, breast milk provides bacteria, antibodies (primarily IgA), and effector lymphocytes that specifically respond to immunological challenges previously encountered by the mothers [5–8].

Among the lymphocytes in breast milk, CD4+ T cells are the most abundant, exhibiting phenotypes associated with mucosal tropism and memory. It has been hypothesized that these cells are transported via an enteromammary pathway and correspond to the immunological experiences acquired from the maternal mucosa [9–11]. Conventionally, CD4+ T cells differentiate into effector cells (Th1, Th2, or Th17); however, they can also acquire a tolerogenic phenotype such as regulatory T (Treg) cells, which maintain a suppressive environment against innocuous antigens through various mechanisms, including the expression of inhibitory molecules such as cytotoxic T lymphocyte antigen (CTLA)‐4 and programed death (PD)‐1 (also recognized as CD152 and CD279, respectively), as well as the production of immunomodulatory cytokines such as transforming growth factor (TGF)‐β and interleukin (IL)‐10 [12–16]. In a recent study, we identified T helper cells in human colostrum that phenotypically correspond to classical memory Treg cells (CD4+CD127‐CD25++CD45RO+Foxp3+) [17].

Treg cells play a central role in regulating the balance between immune activation and tolerance, thereby maintaining immunological homeostasis [18, 19]. Lactation represents a key route for maternal immune transfer, through which Treg cells can be transferred to the offspring, potentially shaping the Treg cell setpoint during a critical neonatal window; this effect persists into adulthood and may extend across generations, with increased frequency, stability, and suppressive capacity associated with a reduced risk of allergic, autoimmune, and inflammatory diseases [20–22]. Nevertheless, beyond the contribution of maternal Treg cells transferred via breast milk, other bioactive components in colostrum support neonatal immune development by promoting Treg cell expansion and functional maturation [23]. These components, together with maternal Treg cells transferred through breastfeeding, may act as part of a coordinated immune network. Breastfed infants exhibit a larger thymus and a higher proportion of Treg cells compared with formula‐fed counterparts, as well as reduced proliferative T‐cell responses to noninherited maternal antigens [24–26]. Collectively, these observations support a role for postnatal nutrition in establishing sustained Treg‐mediated immune regulation.

Another key breast milk component supporting neonatal immunity is secretory IgA, which maintains mucosal homeostasis by neutralizing pathogens and preventing their adherence to epithelial surfaces. Furthermore, IgA actively shapes the mucosal immune environment by influencing T‐helper cell differentiation to pathogens or innocuous antigens [27–31]. In addition to supporting immunomodulatory environments, Treg cells mediate, both directly and indirectly, the production and specialization of antibodies. Thus, the expression of CTLA‐4 and PD‐1 in T cells negatively controls the secretion of immunoglobulins, while TGF‐β promotes the IgA class of B cells and the production of IgA [32–35]. The Treg‐IgA axis emerges as a key regulatory pathway, particularly in the intestinal and mammary mucosa. This interplay is essential for establishing immune tolerance to the microbiota and innocuous antigens and may be especially critical during early life development [27]. The establishment of a symbiotic relationship between the host and the intestinal microbiota relies on appropriate mucosal T‐cell responses to commensal antigens, a process that is strongly influenced during early life by maternal antibodies. While IgG is primarily associated with passive protection against pathogens, IgA plays a central role in mediating host‐commensal interactions at mucosal surfaces. Accordingly, among the immunoglobulin isotypes present in breast milk, IgA may preferentially interact with Treg cells and commensal bacteria, contributing to immune homeostasis [36, 37].

In addition to providing protection, breast milk is the first source of commensal bacteria, which colonize the mucosa and shape the immune system of breastfed newborns [38]. The bacterial diversity in human breast milk is extensive, predominantly featuring skin‐associated bacteria such as *Staphylococcus* and *Streptococcus*, as well as enteric bacteria like *Bifidobacterium*, *Lactobacillus*, and *Enterococcus* [39, 40]. The origin of enteric bacteria in breast milk is still under debate; it is hypothesized that they are transported from the maternal enteric mucosa to the mammary gland via the enteromammary pathway in late pregnancy to colonize the neonatal gut mucosa by breastfeeding [41].

Vertically transmitted gut bacteria appear to be more persistent and better adapted to the intestinal environment than bacteria acquired from other sources, underscoring the importance of early‐life microbial transfer in microbiome assembly [42]. Beyond colonization dynamics, *Bifidobacterium* species have been shown to preferentially promote Treg cell differentiation compared with other commensals, which are critical for maintaining immune tolerance to microbial and dietary antigens [43, 44]. This effect is likely mediated via bacterial metabolites and interactions with antigen‐presenting cells, highlighting a relatively specific association between *Bifidobacterium* and Treg induction [45]. This microbiota–host interaction contributes to the regulation of inflammatory responses and the establishment of immune homeostasis early in life, thereby reducing susceptibility to immune‐mediated disorders [46, 47].

Accumulating evidence suggests that colostrum contains a complex network of microbial and immunological components with potential functional interactions that contribute to the establishment of tolerogenic responses in neonates. In this context, we hypothesized that maternal Treg cells, transferred through breastfeeding, may serve as the central component of a coordinated biological axis. In the present study, we assessed the association between Treg cells, antibodies, and commensal bacteria in colostrum to determine whether cellular and humoral components operate in an integrated manner. This study provides a descriptive characterization of the immune composition of colostrum from healthy women and examines potential interactions among immune and microbial components involved in human colostrum.

## 2. Methodology

### 2.1. Collection of Samples

The study was reviewed and approved by the Investigation, Biosecurity, and Ethics Committees of the National Institute of Perinatology (INPer) (Project Numbers: 2022‐1‐26 and 2024‐1‐66). Healthy participants who were under recovery care at INPer were invited to participate in the study. After informed consent was obtained, colostrum samples (an average of 0.7 mL from 0 to 5 days after delivery) were obtained manually using the Marmet technique in sterile microcentrifuge tubes. The donors had preterm (34–36.9 weeks of gestation) or full‐term (37–42 gestational weeks) pregnancies. Exclusion criteria included immune deficiencies, metabolic diseases, pregnancy complications, or clinically documented maternal infections based on prenatal records. The colostrum sample was aliquoted into two portions: 500 µL was used for Treg cell characterization and antibody quantification, and 200 µL for the extraction of bacterial DNA.

### 2.2. Cell Isolation From Colostrum

Samples were diluted (1:4) in sterile PBS 1x and centrifuged at 600 × *g* for 15 min at room temperature. The supernatant was aliquoted and stored at −20°C for antibody quantification, and the cell pellet was washed twice with 500 µL of sterile PBS 1x. Cell count and viability were assessed using an automated cell counter (Countess Invitrogen, USA) using trypan blue stain (0.4%) (Life Technologies, USA).

### 2.3. Flow Cytometry Treg Cells Identification and Characterization

Colostrum Treg cells were quantified by flow cytometry, following previously described protocols [17]. From each sample, 500,000 live cells per sample were stained for surface or intracellular characterization. Surface immunostaining was used to evaluate the relative expression of CD152 (CTLA‐4) and CD279 (PD‐1), whereas intracellular staining was performed to assess TGF‐β and IL‐10 expressions.

#### 2.3.1. Surface Immunostaining

In the absence of light, total cells were incubated with titrated volumes of fluorochrome‐conjugated monoclonal antibodies and viability dye consisting of: CD45/Pacific Blue (Cat 304029, BioLegend, USA), Fixable and Viability Dye (FVD)/eFluor506 (Cat 65‐08‐66‐14, eBiosciences, USA), CD3/PerCp (Cat 347344, BD Biosciences, USA), CD4/APC‐Cy7 (Cat 3600518, Biolegend, USA), CD45RA/PE‐Texas Red (Cat MHCD45RA17, Invitrogen, USA), CD45RO/FITC (Cat 555492, BD Biosciences, USA), CD25/PE‐Cy7 (Cat 356108, Biolegend, USA), CD152(CTLA‐4)/APC (Cat 349908, Biolegend, USA), and CD279 (PD‐1)/PE (Cat 329906, Biolegend, USA). Fluorescence minus one (FMO) controls were included. Surface‐stained cells were then fixed using 1× FACS Lysing Solution (1:10 dilution; BD Biosciences, San Jose, CA, USA) for 10 min at room temperature in the absence of light. Subsequently, FACS Flow was added to wash excess antibodies, and the samples were centrifuged at 400 × *g* for 5 min. Finally, samples were analyzed by flow cytometry using a BD FACSAria III (DIVA software) instrument equipped with 405, 488, and 633 nm lasers.

#### 2.3.2. Intracellular Immunostaining

Following surface staining with anti CD3, CD4, CD25, CD45RO, and CD45RA antibodies for 15 min at room temperature using the antibody panel described above, cells were washed twice with cell staining buffer (Cat 420201, BioLegend, USA) and subsequently fixed and permeabilized using Cytofix/Cytoperm (Cat. No. 51‐2090KZ, BD Biosciences, San Jose, CA, USA) for 20 min at 4°C. Cells were then washed with Perm/Wash buffer (1:10 dilution; BD Biosciences, Cat. No. 421002), followed by intracellular staining with TGF‐β/PE (Cat. No. 5141127093, MACS) and IL‐10/BV421 (Cat. No. 564053, BD Biosciences) antibodies for 30 min at 4°C. Finally, cells were washed to remove excess antibodies prior to flow cytometric analysis. Finally, samples were acquired on a BD FACSAria III flow cytometer and analyzed using BD FACSDiva software (version 8.0.2).

Expression levels of CD152, CD279, TGF‐β, and IL‐10 were reported as mean fluorescence intensity (MFI), and absolute Treg cell counts were estimated by relating flow cytometry percentages to total cell counts per sample. A detailed description of the gating strategy for Treg identification is provided in the Supporting Information.

### 2.4. Antibody and Cytokine Quantification

Previously thawed and diluted colostrum supernatants were analyzed to quantify antibody isotypes and cytokines using the LEGENDplex Human Immunoglobulin Isotyping Panel (Cat. 740646; BioLegend) and the Human Th Cytokine Panel (Cat. 740001; BioLegend), according to the manufacturer’s instructions. IgA concentrations were measured using the Human Immunoglobulin Flex Set System (BD Biosciences) following a similar protocol. Samples were incubated with capture beads, detection antibodies, and Streptavidin‐PE and analyzed on a BD FACSAria III flow cytometer and analyzed using BD FACSDiva software (version 8.0.2). Standard curves were generated using log‐transformed data fitted to a four‐parameter logistic model for the isotyping panel and a five‐parameter model for IgA. Final concentrations were calculated by interpolation from the respective standard curves. A detailed description of the assay procedures and standard curve models is provided in the Supporting Information.

### 2.5. Isolation and Quantification of Bacterial DNA From Colostrum

Total DNA was extracted from colostrum samples using the QIAamp DNA Mini Kit (Cat. 51306, QIAGEN, Hilden) and quantified with a NanoDrop spectrophotometer (Thermo Scientific). To enhance detection sensitivity, DNA from total bacteria and five specific genera (*Bifidobacterium*, *Lactobacillus*, *Staphylococcus*, *Streptococcus*, and *Enterococcus*) were preamplified by conventional PCR using genus‐specific primers, as listed in Table 1. Quantitative PCR (qPCR) was subsequently performed using the SYBR Green PCR Master Mix system (QIAGEN, Cat. 204001) with standard curves generated from both representative samples and ATCC reference strains for each bacterial genus: *Bifidobacterium longum* subsp. *Infantis* (Reuter) Mattarelli et al. (ATCC. No. 15697D‐5), *Lactobacillus acidophilus* (Moro) Hansen and Mocquot (ATCC, No. 4357D‐5), *Staphylococcus epidermidis* (Winslow and Winslow) Evans (ATCC, No. 12228D‐5), *Streptococcus agalactiae* Lehmann and Neumann (ATCC, No. BAA‐611D‐5), *Enterococcus faecium* (Orla‐Jensen) Schleifer and Kilpper‐Balz (ATCC, No. BAA‐472D‐5). Genus‐specific DNA concentrations were calculated by interpolating Ct values against the corresponding standard curves and were expressed per 100,000 pg of total DNA. Detailed methodology is provided in the Supporting Information.

**Table 1:** PCR conditions for bacterial DNA amplification.

Bacteria genus	Primers (5´ to 3´)		Temperature (°C)	Amplified size (pb)
Universal bacterial primers (16S rRNA)	Forward	AGAGTTTGATCCTGGCTCAG	60	500
	Reverse	GGCTGCTGGCACGTAGTTAG		
*Bifidobacterium*	Forward	GATTCTGGCTCAGGATGAACGC	60	245
	Reverse	CTGATAGGACGCGACCCCAT		
*Lactobacillus*	Forward	AGCAGTAGGGAATCTTCCA	60	341
	Reverse	CACCGCTACACATGGAG		
*Enterococcus*	Forward	CCCTTATTGTTAGTTGCCATCATT	61	144
	Reverse	ACTCGTTGTACTTCCCATTGT		
*Staphylococcus*	Forward	GGCCGTGTTGAACGTGGTCAAATCA	60	370
	Reverse	TIACCATTTCAGTACCTTCTGGTAA		
*Streptococcus*	Forward	GTACAGTTGCTTCAGGACGTATC	61	197
	Reverse	ACGTTCGATTTCATCACGTT		


*Note:* Primer sequences used for amplification of the 16S rRNA subunit, based on the data reported by (Khodayar‐Pardo et al. [39]). PCR and qPCR conditions for each of the bacterial genera analyzed are also provided.

### 2.6. Statistical Analysis

The normality of continuous variables was assessed using the Shapiro–Wilk test. Group comparisons were conducted using the Mann–Whitney *U* or Student’s *t*‐test for continuous variables and chi‐square or Fisher’s exact test for categorical variables. Associations among Treg cell frequencies, immunoglobulin and cytokines levels, and bacterial genera were evaluated using Spearman’s rank correlation, adjusted using the Holm–Bonferroni method. A correlation matrix was visualized with the corrplot R package to explore interaction patterns. Principal component analysis (PCA) based on Spearman correlations was performed with the FactoMineR and factoextra packages to reduce dimensionality and identify key sources of variance. Variable contributions to PCA clustering were validated through multivariate analyses in SPSS. Statistical significance was set at *p*  < 0.05 (two‐tailed).

## 3. Results

### 3.1. Clinical Characteristics of Donors and Immune Components in Colostrum

This study included 33 donors, whose information is summarized in Table 2. Participants were within the reproductive age range, had uncomplicated pregnancies, and presented an average BMI of 25 ± 6.9 kg/m^2^. They had between one and three previous pregnancies, and all children were breastfed. The mean gestational age at birth was 37.4 ± 1.6 weeks, and about two‐thirds of the infants were born at term with appropriate growth for gestational age. Most of the deliveries were cesarean sections (73%). All infants had healthy Apgar scores at one and 5 minutes after birth, and their birth weights ranged from 2.5 to 4.0 kg with lengths between 46 and 51 cm. Female newborns represented the predominant sex in the cohort.

**Table 2:** Demographic data of the volunteer mothers and their newborns.

Parameter	Measure
Mother (*n* = 33)	
Age (years)	26.1 ± 8.3
Pregestational BMI (Kg (m)^2^)	25 ± 6.9
N° gestation	2.2 ± 1.1
Newborn	
Gestational age (weeks)	37.4 ± 1.6
Preterm (34–36.9 weeks)	13 (39%)
Term (37–42 weeks)	20 (61%)
Birth weight (kg)	3.49 ± 3.9
Lenght (cm)	48.0 ± 2.2
Apgar score	8/9 ± 1.4/1.2
Delivery mode	
Vaginal birth (*n*)	9 (27%)
Cesarean section (*n*)	24 (73%)
Gender	—
Female (*n*)	18 (55%)
Male (*n*)	15 (45%)

*Note:* Mean values and standard deviations are presented.

The composition of immune cells in colostrum varied significantly between donors (Table 3). On average, the 33 samples contained 4447 ± 8619 Treg cells/mL, representing 25% ± 13% of the total helper T cells. Table S1 also presents the concentration of Treg cells per mL subclassified by the collection day. The highest number of cells was observed within the first days of collection (day 1:28125 ± 23666 cells/mL), followed by a progressive decline until the fifth postpartum day. The predominant immunoglobulin was IgA, with an average concentration of 2207.6 × 10^3^ pg/mL, followed by IgG3 (1121.3 × 10^3^ pg/mL), IgG1 (100.6 × 10^3^ pg/mL), IgG4 (29.6 × 10^3^ pg/mL), IgG2 (10.7 × 10^3^ pg/mL), and IgM (2.6 × 10^3^ pg/mL). IgE antibodies were not detected. Regarding bacterial DNA, the most abundant genera were skin‐associated bacteria, particularly *Staphylococcus* (30 × 10^−3^ fg) and *Streptococcus* (31 × 10^−3^ fg). Gut‐associated genera were less prevalent; among these, *Enterococcus* (2.3 × 10^−3^ fg) was the most enriched, followed by *Lactobacillus* (0.9 × 10^−3^ fg) and *Bifidobacterium* (0.4 × 10^−3^ fg). Together, these data provide a descriptive overview of the immune and microbial composition of colostrum in our cohort of healthy women, highlighting notable interindividual variability. The high abundance of Treg cells during the first postpartum day, the predominance of IgA among antibody isotypes, and the enrichment of skin‐associated bacterial genera underline the dynamic nature of early breast milk.

**Table 3:** Quantification of Treg, antibodies, and bacteria in colostrum.

Composition	Measure
Treg	
Treg (cell/mL)	4447 ± 8619 (49–44859)
Treg‐p (Treg percentage among T helper cells)	25.0 ± 13.0 (5.0–53.0)
Immunoglobulins	
IgG1 (×10^3^ pg/mL)	100.6 ± 147.8 (0.184–531.1)
IgG2 (×10^3^ pg/mL)	10.7 ± 12.5 (1.2–58.8)
IgG3 (×10^3^ pg/mL)	1121.3 ± 1730.5 (2.0–8705.4)
IgG4 (×10^3^ pg/mL)	29.6 ± 44.4 (0.6–186.6)
IgM (×10^3^ pg/mL)	2.6 ± 1.5 (2.0–7.3)
IgA (×10^3^ pg/mL)	2207.6 ± 1067.6 (322.0–5021.0)
Bacteria genus^a^	
DNA universal (×10^−3^ fg)	48.0 ± 180.0 (0.1–990.0)
DNA *Bifidobacterium* (×10^−3^ fg)	0.4 ± 0.8 (0.0–4.3)
DNA *Lactobacillus* (×10^−3^ fg)	0.9 ± 1.8 (0.0–9.3)
DNA *Enterococcus* (×10^−3^ fg)	2.3 ± 1.0 (0.0–44.0)
DNA *Streptococcus* (×10^−3^ fg)	31.0 ± 76.0 (0.0–320.0)
DNA *Staphylococcus* (×10^−3^ fg)	30.0 ± 110.0 (0.0–480.0)

*Note:* For each parameter, the mean and standard deviation are presented. The minimum and maximum values are shown in parentheses.

^a^In the case of bacteria, concentrations are expressed per 100,000 pg of total DNA.

To capture temporal dynamics during early lactation, Table S1 summarizes antibody, Treg cell, and bacterial profiles in colostrum stratified by the collection day. Overall, the results show that Treg cell counts were highest during the first postpartum day and declined thereafter, while Treg cell proportions among CD4^+^ T cells remained relatively stable across days. IgA was the predominant immunoglobulin throughout the colostrum period, whereas IgG subclasses exhibited a progressive increase over time. Skin‐associated bacterial genera predominated across all days, while gut‐associated genera were consistently detected at lower abundances with marked interindividual variability. Overall, the collection day within the first five postpartum days had no evident impact on the immune or microbial composition of colostrum samples.

### 3.2. Relationship Between Treg Cells, Immunoglobulin, and Commensal Bacteria

**Figure 1:**
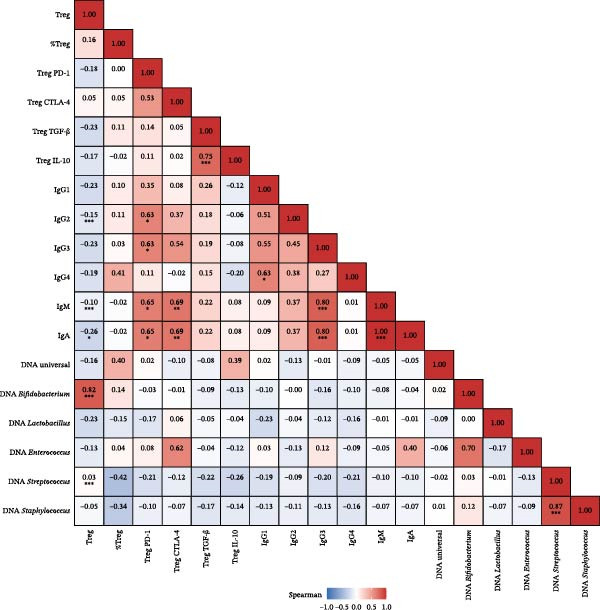
Correlation matrix between Treg, cell membrane markers, immunoglobulins, and bacterial DNA in colostrum. Positive correlations are displayed in red, while negative correlations are shown in blue. The intensity of the color corresponds to the strength of the correlation coefficient. The values shown in the matrix represent Pearson correlation coefficients.  ^∗^ *p*  < 0.05,  ^∗∗^ *p*  < 0.001, and  ^∗∗∗^ *p*  < 0.0001. BMI, body mass index before pregnancy; BW, birth weight; DM, delivery mode; HC, baby’s head circumference; Treg (cells/mL); %Treg, Treg percentage among T helper cells.

To elucidate the interrelationships between cellular and soluble components in colostrum, we conducted bivariate Spearman correlation tests involving Treg cells, immunoglobulins, and the abundance of DNA from bacteria (Figure 1).

Our analysis indicated that the quantities and percentages of Treg cells do not correlate with the own expression levels of their immunoregulatory molecules, CTLA‐4 and PD‐1. Significant correlations were observed among secretory molecules (TGF‐β and IL‐10, *r* = 0.75) as well as among surface markers (CTLA‐4 and PD‐1, *r* = 0.53).

Regarding Treg cell counts, a negatively correlated with IgA levels (*r* = −0.26), IgM (*r* = −0.10), and IgG2 (*r* = −0.15) isotype; however, these associations were not statistically significant. Remarkably, Treg cells showed a strong positive correlation with *Bifidobacterium* (*r* = 0.82), while no significant correlations were observed with other bacterial genera.

Additionally, PD‐1 expression in Treg cells was positively correlated with IgG2 and IgG3 (*r* = 0.63 for both). IgA and IgM correlated positively with PD‐1 y CTLA‐4 (*r* = 0.65 and 0.69, respectively), but these markers were not significantly related to total bacterial counts or specific bacterial genera. Immunoglobulins showed interrelated patterns; IgG1 was highly correlated with IgG4 (*r* = 0.63). IgG3 concentration correlates with IgA and IgM (*r* = 0.80). Among bacterial relationships, a strong positive correlation was observed between *Streptococcus* and *Staphylococcus* (*r* = 0.87), with no correlation found between other bacterial genera or total bacterial levels.

To explore the factors that influence the immune profile of colostrum, we quantified Th cytokines concentrations and integrated them into the correlation analysis. As shown in the Supporting Information (Figure S2), specific cytokines were grouped into positively associated clusters with Treg cell frequencies, their expression of immunoregulatory molecules, and antibody concentrations. Effector cytokines (IL‐13, IL‐2, IFN‐γ, IL‐17 F, and IL‐4) showed positive associations with CTLA‐4 and PD‐1 expression in Treg cells; however, these associations were not supported by statistically significant evidence. In contrast, IgG3, IgM, and IgA exhibited significant clusters of positive associations with the same set of cytokines.

**Figure 2:**
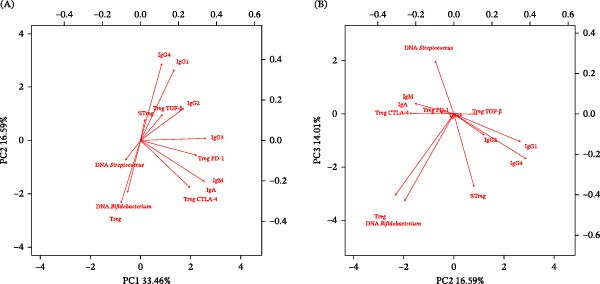
Principal component analysis (PCA) of Treg, cell markers, bacterial DNA, and immunoglobulins. Data represents the variables with significant loadings. (A) The biplot of PC1 and PC2 explains 33.46% and 16.59% of the total variance, respectively. The variables cluster into four main groups: the first group includes Treg, *Bifidobacterium*, and *Streptococcus* DNA; the second group consists of CD152, CD279, IgG3, IgA, and IgM; the third group is composed of IgG1, IgG2, IgG4, TGF‐ β, and Tregp. (B) The biplot of PC2 and PC3 accounts for 16.59% and 14.01% of the total variance, respectively. BMI, body mass index; CD152 (CTLA‐4); CD279 (PD‐1); MD, mode of delivery; %Treg, Treg percentage among T helper cells.

PCA was performed to further explore the relationships among variables. The first three principal components accounted for 64.06% of the total variance. The biplot of PC1 and PC2 (Figure 2A) revealed three main clusters of variables, indicating positive associations among variables within each cluster. The first cluster comprised Treg cells along with DNA from *Bifidobacterium* and *Streptococcus*. The second cluster included Treg cell‐associated receptors (CTLA‐4 and PD‐1) together with immunoglobulins (IgA, IgM, and IgG3). The third cluster consisted of IgG1, IgG4, and IgG2, along with TGF‐β and the percentage of Treg cells. In contrast, the first and third clusters were oriented in opposite directions, suggesting negative associations between their respective variables. Interestingly, the biplot of PC2 and PC3 showed a predominant clustering of Treg cells and DNA from *Bifidobacterium* within the same group (Figure 2B).

Altogether, these findings may support the presence of a coordinated network involving Treg cells and specific bacterial genera in colostrum. The observed positive association between Treg cells and *Bifidobacterium* may suggest a potential immunoregulatory link during early lactation. Moreover, the clustering of immune markers and microbial variables identified through multivariate analysis may reflect an interplay between maternal immune factors and microbial components, which could contribute to neonatal immune priming and tolerance induction through breastfeeding.

## 4. Discussion

Breast milk is the primary source of passive immunity transferred from the mother to infant after birth. This protective function is attributed to the presence of antibodies, leukocytes, such as T cells, which can play a critical role in transferring specific immune memory, and commensal bacteria that contribute to immune development in the newborn. Among these components, maternal Treg cells represent a memory‐phenotype population that may contribute to neonatal immune maturation and tolerance following transfer through breastfeeding. Based on this, we hypothesized that maternal components may be interrelated and function as part of a coordinated immune microbial network shaping the neonatal gut microbiota and immune system. In this study, we quantified and characterized the phenotype of Treg cells, assessed immunoglobulin profiles, and determined the predominant bacterial genera in colostrum. Furthermore, we evaluated the association among these components to explore the potential transfer of this maternal immune microbial network to the breastfed neonate.

The cohort of milk samples evaluated in this study shows that the composition of breast milk colostrum varied significantly between donors. For example, the Treg cell counts constitute a significant proportion of CD4+ T cells in colostrum. The composition of breast milk was observed to adapt in response to different factors, supporting the needs of the infant based on characteristics such as gestational age, birth mode, and newborn gender, even if the mother or newborn has an infection [48, 49]. The results of this study revealed that the number of Treg cells decreased after the first day postpartum (Table S1). However, maternal and newborn factors, such as prepregnancy BMI, delivery mode, birth weight, and head circumference, did not significantly influence the presence of Treg cells. This study did not consider the microenvironment in which the cells develop, which may further influence the presence of Treg cells. Moreover, other regulatory cell subpopulations that could be modulated by maternal factors were not evaluated.

Characterization of colostrum demonstrated a predominant enrichment of *Staphylococcus* and *Streptococcus* (Table 3), bacterial genera commonly found on the skin and in breast milk [50]. These genera exhibit a homogeneous distribution throughout the mammary gland, which may explain the positive correlation observed between them (Figure 1). Their presence may be critical for newborn colonization; in particular, *Staphylococcus* has been reported to generate de novo Treg cells [51]; however, in this study, no associations were observed in the correlation matrix or the PCA analysis (Figures 1 and 2). In contrast, *Lactobacillus* and *Bifidobacterium* are less predominant in colostrum (Table 3) [52, 53]; despite this, the correlation matrix shows a positive relationship between Treg cell abundance and the presence of *Bifidobacterium* in colostrum (Figure 2A,B). This possible association may be related to the ability of *Bifidobactrerium* species to promote the differentiation of Treg cells at mucosal sites through the secretion of metabolites (mainly butyrate and acetate) [54]. Maternal Treg cells, which may originate from the intestinal mucosa, could retain functional imprinting from their site of activation, influencing their subsequent interactions with specific commensal bacteria, such as *Bifidobacterium*, known to play a key role in intestinal immune regulation. Although breast milk also contained significantly more T cells with a broad homing profile, other lymphocyte subpopulations (for example, cytotoxic T cells) resident in the mammary tissue [55] may be transferred as well, potentially associating with bacterial genera like *Staphylococcus* and *Streptococcus*, which also inhabit the mammary environment. Nevertheless, there could be other populations of regulatory immune cells that could be potentially associated with commensal bacteria, suggesting an alternative perspective for further exploration.

B lymphocyte trafficking to the mammary gland likely contributes to the immunoglobulin composition of milk [10, 56]. Breast milk is highly enriched with immunoglobulins, IgA being the most abundant immunoglobulin in colostrum, as shown in this study (Table 3) [49, 57]. The IgG3 predominance observed in this cohort (Table 3) might reflect differences in antigenic exposure among Mexican women compared to those found in other regions, such as southern Latin America and Europe [49, 58]. Contrary to our expectations, no significant associations were observed between Treg cells or bacterial genera and any immunoglobulin isotype, including IgA, in colostrum (Figures 1 and 2). However, murine models have reported that breast milk antibodies, specifically IgG and IgA, limit the activation of B and T lymphocytes to vaccine and commensal bacteria by neutralization, preventing direct exposure and allowing the offspring to develop their own defense mechanisms [31, 37]. It is important to note that differences in immunoglobulin subclass composition between mice and humans may underlie these divergent findings: mice express four IgG subclasses (IgG1, IgG2a, IgG2b, and IgG3) [59], whereas humans express four distinct IgG subclasses (IgG1, IgG2, IgG3, and IgG4). Although orthologous genes are present between species, these interspecies differences may underlie the absence of IgA and IgG associations observed in our study, suggesting that findings from murine models may not fully reflect the complexity of human humoral immune responses. Furthermore, although this study only evaluated total IgA, previous research has suggested that maternal IgA2 is preferentially associated with genera such as *Bifidobacterium*, *Pseudomonas*, *Lactobacillus*, and *Paracoccus*, highlighting a potential role for this subclass in shaping the neonatal microbial composition and representing a limitation of our analysis [60].

Several studies indicate that immunomodulatory cytokines present in breast milk can influence the neonatal gut microbial composition and immune maturation. For instance, higher concentrations of TGF‐β in breast milk have been associated with increased richness and diversity of the infant gut microbiota, potentially modulating early microbial communities in breastfed neonates [61]. Cytokines such as TGF‐β and IL‐10 are also implicated in promoting mucosal immune tolerance, enhancing IgA class switching, and supporting epithelial integrity, processes that indirectly shape microbial colonization patterns [62]. In our study, we did not observe significant associations between the major cytokines produced by Tregs and those present in colostrum with either antibodies or bacterial abundances. These results suggest that while breast milk cytokines contribute to early immune programing, their relationships with humoral, microbial, and cellular components may be influenced by additional factors, highlighting the complexity of these interactions.

PD‐1 has been shown to mediate T‐cell inhibition, and it has been associated with an increase in IgM memory B cells [63], which potentially reflects an indirect mechanism for controlling T‐cell proliferation. Although CTLA‐4 plays a distinct role in regulating immune responses, it primarily controls T‐cell proliferation early in immune responses, mainly within lymph nodes, while PD‐1 functions later, primarily in peripheral tissues [64]. Positive associations were observed between CTLA‐4 expression and immunoglobulins IgA and IgM (Figure 1), which could suggest that these receptors may indirectly influence the regulation of the Treg–bacteria–immunoglobulin axis. There is an ongoing debate regarding whether CTLA‐4 deficiency leads to increased or decreased B‐cell activation and immunoglobulin production [65, 66]. Nevertheless, these receptors, expressed on Treg cells, are likely essential to modulate the secretion of antibody subclasses, which affects bacterial coating and immune tolerance.

Some limitations of this study include the small sample size, its descriptive cross‐sectional design, and the assessment of only total IgA and selected immunoglobulin subclasses, which may have overlooked associations with other isotypes. Regulatory immune populations beyond Treg cells were not evaluated, and microbial profiling was limited to the genus level. In addition, Treg cells were identified based on surface phenotypic characteristics without the inclusion of FoxP3 or CD127 markers. While this approach has been applied in previous studies of human colostrum and breast milk and allows robust quantitative and comparative analyses, it may limit the resolution of specific Treg subsets. Despite these limitations, the use of human samples with homogeneous characteristics helped reduce the variability and strengthened the internal consistency of the findings.

## 5. Conclusion

This study describes a coordinated cellular and microbial network of human colostrum, highlighting a consistent association between Treg cell abundance and the presence of *Bifidobacterium*. These findings define a reproducible immune‐bacteria relationship in early breast milk, without evidence of association with immunoglobulin profiles.

## Funding

This study was funded by the National Institute of Perinatology (INPer), Mexico (Grants 2022‐1‐26 and 2024‐1‐66).

## Conflicts of Interest

The authors declare no conflicts of interest.

## Supporting Information

Additional supporting information can be found online in the Supporting Information section.

## Supporting information


**Supporting Information** Supporting Methodology: 1. Antibody and cytokines quantification. 2. Extraction of total DNA in human colostrum samples. 3. Quantification of total bacterial DNA and the genera *Bifidobacterium*, *Lactobacillus*, *Enterococcus*, *Staphylococcus*, and *Streptococcus*. 4. Statistical analysis. Figure S1. Gating strategy for the selection of Treg cells in colostrum. Figure S2. Correlation matrix between population characteristics, Treg cells, cell membrane markers, immunoglobulins, and bacterial DNA present in colostrum. Table S1. Treg cells, antibodies, and bacteria in colostrum samples by day.

## Data Availability

The data that support the findings of this study are available from the corresponding author upon reasonable request.
