# Supplementary material for: Coordination Between Treg Cells and Bifidobacterium in the Immune–Bacterial Network of Human Colostrum
Source: J Immunol Res. 2026 Jun 22;2026:8677501. doi: 10.1155/jimr/8677501 (PMC13287830; doi:10.1155/jimr/8677501)
Supplement: Supplementary file 1 — Supporting Information Supporting Methodology: 1. Antibody and cytokines quantification. 2. Extraction of total DNA in human colostrum samples. 3. Quantification of total bacterial DNA and the genera Bifidobacterium, Lactobacillus, Enterococcus, Staphylococcus, and Streptococcus. 4. Statistical analysis. Figure S1. Gating strategy for the selection of Treg cells in colostrum. Figure S2. Correlation matrix between population characteristics, Treg cells, cell membrane markers, immunoglobulins, and bacterial DNA present in colostrum. Table S1. Treg cells, antibodies, and bacteria in colostrum samples by day. [file JIMR-2026-8677501-s001.pdf]

SUPPLEMENTARY METHODOLOGY

1. *Antibody and cytokines quantification*

Previously stored and diluted colostrum supernatants were thawed, and the concentrations of antibody isotypes (IgG1, IgG2, IgG3, IgG4, IgM, and IgE) were determined using the LEGENDplex™ Human Immunoglobulin Isotyping Panel (Cat. 740646; BioLegend). In the same samples, cytokine levels were assessed using the LEGENDplex™ Human Th Cytokine Panel (Cat. 740001; BioLegend). Both assays were performed following the manufacturer's instructions. Briefly, 5 µL of the sample was incubated for 2 hours with 5 µL of capture beads and 10 µL of Buffer solution. After incubation, beads were washed and centrifuged at 200 xg for 5 min, then 5 µL of detection biotinylated antibodies were added and incubated for 1 hour. Subsequently, the beads were washed and incubated for 30 min with Streptavidin-PE. A final wash was performed, and the samples were reconstituted in the wash buffer. To quantify IgA, samples were evaluated using the Human Immunoglobulin Flex Set System (Cat 558681, BD Biosciences, USA), and the same methodology was used as described above. All samples were analyzed using a FACS ARIA III cytometer with DIVA V8.0.2 software (BD Biosciences). Log-transformed data were used to obtain standard curves fitted to 8 discrete points using a four-parameter logistic model for the Immunoglobulin Isotyping Panel, while we used a standard curve fitted to 10 discrete points using a five-parameter logistic model for the IgA Flex Set System. Concentrations were calculated by interpolation of the corresponding standard reference curves.

2. *Extraction of total DNA in human colostrum samples.*

Total DNA was extracted from colostrum samples using the QIAamp® DNA Mini Kit (Cat. 51306, QIAGEN, Hilden). Briefly, 200 µL of colostrum was treated with 20 µL of Proteinase K (Cat. 19157, QIAGEN) for 1 min and 200 µL of buffer AL®. Then, the total volume was then transferred to the QIAamp silica-based membrane and washed twice with buffer AW1 and AW2, respectively. Finally, the DNA was eluted in 200 µL of AE buffer (QIAGEN Protease). The DNA (80 µL) was concentrated by mixing extracted DNA with 40 µL of Ammonium Acetate 7.5 M, 160 µL of molecular biology grade ethanol and 6.4 µL of Glycogen 20ng/µL. After 30 minutes at -70°C of incubation, samples were centrifuged at 15300 g for 15 minutes at 4°C and the pellet was washed twice with Ethanol 70%. After drying, DNA was resuspended in 30 µL of molecular biology grade water, and the total DNA quantification was accomplished using a UV-visible spectrophotometer, NanoDropTM (Thermo Scientific).

3. *Quantification of total bacterial DNA and the genera Bifidobacterium, Lactobacillus, Enterococcus, Staphylococcus and Streptococcus.*

In order to improve the quantification sensitivity of DNA extracted from colostrum samples, total bacteria DNA (universal), as well as DNA from *Bifidobacterium*, *Lactobacillus*, *Staphylococcus*, *Streptococcus* and *Enterococcus* were pre-amplified by conventional PCR using a Mastercycler gradient Thermal cycler (Eppendorf Scientific, USA). To accomplish this, 100,000 pg of concentrated DNA was mixed with 12.5 µL of Taq PCR Master Mix 2X (QIAGEN, Cat. 201443), 0.125 µL of each primer set reported in previous work (Table 1) and molecular biology grade water to get a final volume of 25 µL of reaction. Then, the DNA pre-amplification conditions of universal bacteria DNA, *Bifidobacterium*, *Lactobacillus, Staphylococcus* and *Sreptococcus* consisted of a pre-denaturalization at 95 °C for 2 min, followed by 15 cycles of 95° C for 30 s (denaturalization), 60°C for 30 s (alignment) and 72°C for 30 s (extension). For *Enterococcus*, the PCR protocol included an initial pre-denaturation at 95 °C for 2 min, followed by 20 cycles of 95° C for 30 s (denaturation), 61°C for 30 s (annealing), and 72°C for 30 s (extension).

Subsequently, the DNA concentration of total bacteria and individual bacterial genera was determined using quantitative PCR (qPCR). To achieve this, a representative colostrum sample was first used to generate standard curves for the six bacterial genera, using the following concentrations: 100 ng/µL, 10 ng/µL, 1 ng/µL and 10 pg/µL. The standard curve samples were amplified using the *SYBR Green PCR Master Mix system* (QIAGEN, Cat. 204001), 5 µL of *DNA polymerase enzyme HotStarTaq Plus* (QIAGEN, Cat. 203605), 0.125 µL of each primer set (Table 1) and 2.75 µL of molecular biology grade water. The qPCR was performed with the 75-well Rotor-Gen® Q (QIAGEN). The applied thermal protocol consisted of 3 stages: enzyme activation at 95 °C for 10 min, denaturation at 95 °C for 20 s, an annealing temperature of 60 °C for 15 s was used for all genera, except for *Enterococcus*, which required 61 °C, and extension at 72 °C for 20 s, for 40 cycles. The melting curve was performed from 72 °C to 95 °C, with temperature increases of 1 °C every 5 seconds to ensure a single product of amplification. The Threshold for each gen was determined with the *Rotor-Gene RealTime Analysis Software*.

Once the standard curve was standardized, it was replicated with the DNA of ATCC strains of each of the genera of interest: *Bifidobacterium longum subsp. Infantis* (Reuter) Mattarelli et al. (ATCC. No. 15697D-5), *Lactobacillus acidophilus* (Moro) Hansen and Mocquot (ATCC, No. 4357D-5), *Staphylococcus epidermidis* (Winslow and Winslow) Evans (ATCC, No.12228D-5), *Streptococcus agalactiae* Lehmann and Neumann (ATCC, No.BAA-611D-5), *Enterococcus faecium* (Orla-Jensen) Schleifer

and Kilpper-Balz (ATCC, No. BAA-472D-5); the same four concentrations (100 ng/µL, 10 ng/µL, 1 ng/µL and 10 pg/µL) were used for the curve and conditions for qPCR. After this, all colostrum samples were amplified with the same qPCR amplification conditions, and the DNA quantification in each sample was calculated by interpolating the Ct values (threshold cycle) in the linear regression equation obtained with the standard curve. The results were reported as genus-specific DNA concentration per 100,000 pg of total DNA.

4. Statistical analysis

Continuous variables were assessed for normality using the Shapiro-Wilk test. For comparisons between groups, bivariate analyses were performed using the Mann-Whitney U test or Student's t-test for continuous variables, and Chi-square or Fisher's exact test for categorical variables, as appropriate. To explore associations among the relative proportions of regulatory T cells (Tregs), serum immunoglobulin and cytokines levels, and bacterial genera, Spearman's rank correlation coefficients were calculated. A correlation matrix based on Spearman's ρ values, adjusted using the Holm–Bonferroni method, was constructed and visualized using corplot (corrplot R package, version 0.92) to identify potential patterns of interaction among immunological and microbial variables. Subsequently, a Principal Component Analysis (PCA) was conducted using the *FactoMineR* and *factoextra R* packages (R version 4.0, RStudio 1.4 or later). Biological variables were pre-selected based on mechanistic plausibility, immunopathological relevance, and suitability for multivariable modeling. Continuous variables were standardized (z-score) prior to PCA to ensure comparability. The analysis used the Pearson correlation matrix, with component retention guided by eigenvalues >1 (Kaiser criterion), scree plot inspection, and cumulative variance explained. Biplots of the first two components were used to visualize observations and variable loadings, facilitating detection of clustering patterns, collinearity, and potential functional axes.

To confirm the relevance of variables contributing to PCA clustering, additional validation was performed using multivariate procedures in SPSS version 25.0 (IBM Corp., Armonk, NY, USA). All statistical tests were two-tailed, and a P value < 0.05 was considered statistically significant.

SUPPLEMENTARY FIGURES

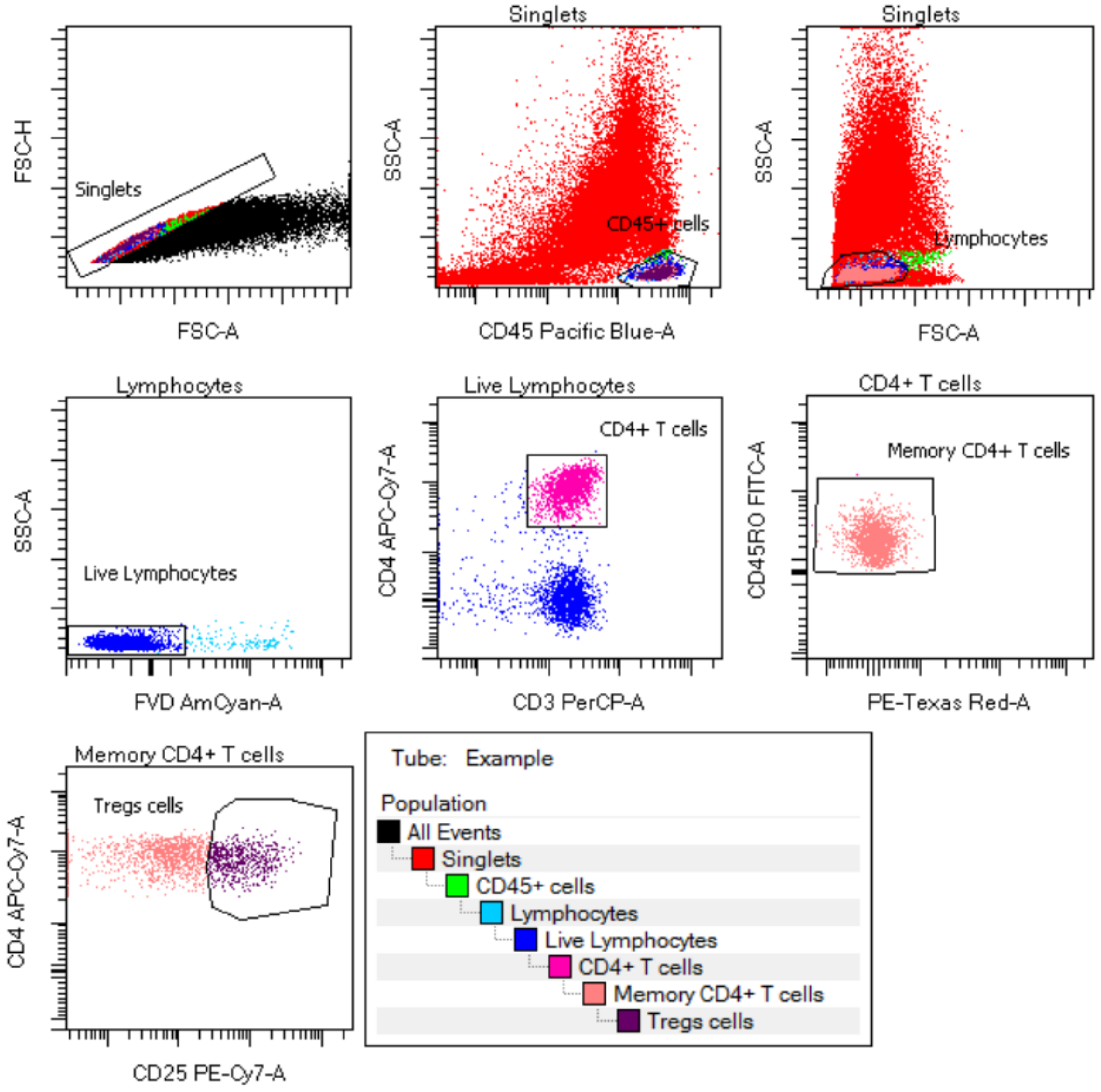

Supplementary Fig 1. Gating strategy for the selection of Treg cells in colostrum. Briefly, singlet cells were first selected based on forward scatter area versus height (FSC-A vs. FSC-H) among total events. From singlets, lymphocytes were identified according to their characteristic light-scatter properties ($CD45^+$ vs. SSC-A). Viable T lymphocytes were then gated by excluding dead cells using the fixable viability dye (FVD^-). Subsequently, $CD3^+CD4^+$ helper T cells were identified. Within the $CD4^+$ T-cell compartment, memory T cells were selected based on $CD45RO^+CD45RA^-$ phenotype. Regulatory T cells were defined as $CD25^{++}$ cells within the memory $CD4^+$ T-cell population.

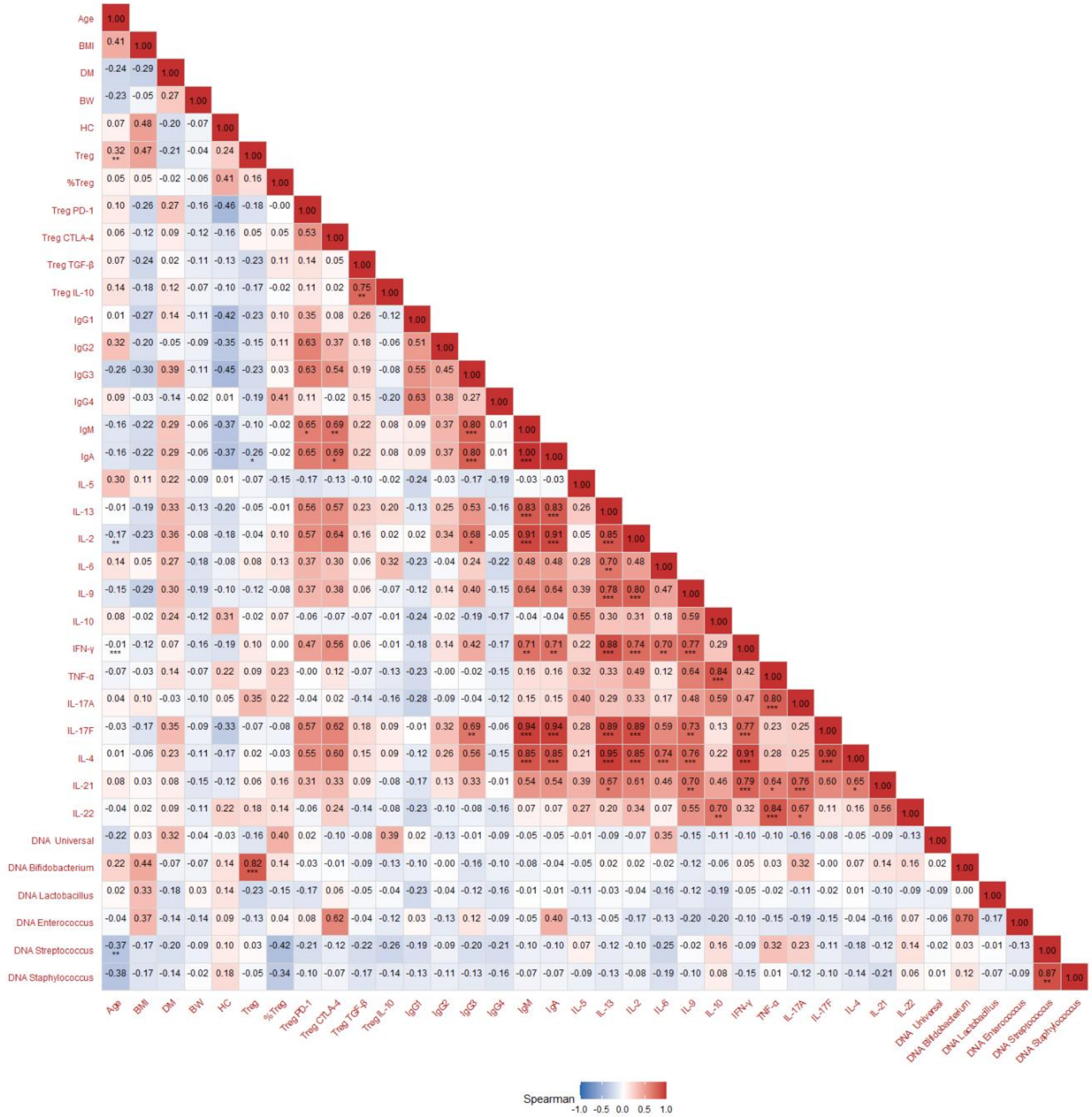

Supplementary Fig 2. Correlation matrix between population characteristics, Treg cells, cell membrane markers, immunoglobulins, and bacterial DNA present in colostrum. Positive correlations are displayed in red, while negative correlations are shown in blue. The intensity of the color corresponds to the strength of the correlation coefficient. The values shown in the matrix represent Pearson correlation coefficients. * = $p < 0.05$, ** = $p < 0.001$, *** = $p < 0.0001$. Body Mass Index before pregnancy (BMI); Delivery mode (DM); Birth weight (BW); Baby's head circumference (HC); Treg (cel/ml); Tregp (Treg percentage among T helper cells).

SUPPLEMENTARY TABLES

Supplementary Tab 1. Treg cells, antibodies, and bacteria in colostrum samples by day

Composition	Measure*
Treg (cel/mL)	4447 ± 8619 (49 – 44859)
Day 1 (cel/mL) (n=2)	28125 ± 23666 (11390 - 44859)
Day 2 (cel/mL) (n=14)	3600 ± 3698 (181 - 8378)
Day 3 (cel/mL) (n=17)	3711 ± 5161 (49 - 15652)
Day 4 (cel/mL) (n=3)	1847 ± 2253 (62 - 5026)
Day 5 (cel/mL) (n=4)	312 ± 136 (135 - 512)
Treg-p (Treg percentage among T helper cells)	25.0 ± 13.0 (5.0 – 53.0)
Day 1 (%)	30 ± 5 (27 - 34)
Day 2 (%)	20 ± 14 (8 - 44)
Day 3 (%)	25 ± 13 (5 - 53)
Day 4 (%)	22 ± 17 (5 - 40)
Day 5 (%)	32 ± 7 (23 - 36)
Treg in preterm newborn (cel/mL)	2752 ± 4613 (62 – 15652)
Treg in term newborn (cel/mL)	5662 ± 10371 (49 – 44859)
Treg in Cesarean section (cel/mL)	5791 ± 10105 (135 – 44859)
Treg in Vaginal birth (cel/mL)	1804 ± 2856 (49 – 8378)
Treg in female newborn	6913 ± 11395 (49 – 44859)
Treg in male newborn	1946 ± 2420 (62 – 8378)
**Immunoglobulins**	
IgG1 (x10^3 pg/mL)	100.6 ± 147.8 (0.184 – 531.1)
Day 1	3.3 ± 4.501 (0.184 – 6.55)

Day 2	18.9 ± 15.6 (1.9 – 41.4)
Day 3	105.9 ± 161.8 (0.4 – 531.1)
Day 4	205.9 ± 180.6 (35 – 395)
Day 5	191.7 ± 168 (20.1 – 186.5)
IgG2 (x10^3 pg/mL)	10.7 ± 12.5 (1.2 – 58.8)
Day 1	4.4 ± 2.8 (2.4 – 6.4)
Day 2	3.9 ± 2.3 (1.9 – 5.4)
Day 3	13.1 ± 15.4 (1.1 – 58.8)
Day 4	11.2 ± 7.3 (4.5 – 19)
Day 5	14.47 ± 13.2 (4.3 – 33.4)
IgG3 (x10^3 pg/mL)	1121.3 ± 1730.5 (2.0 – 8705.4)
Day 1	2 ± 85.7 (43.8 – 59.1)
Day 2	577.4 ± 858 (69.3 – 2439.2)
Day 3	1483 ± 2302 (9.3 – 8705.4)
Day 4	1234.1 ± 863.2 (565 – 2208.6)
Day 5	1080 ± 523.2 (477.5 –1670.1)
IgG4 (x10^3 pg/mL)	29.6 ± 44.4 (0.6 – 186.6)
Day 1	1 ± 0.7 (0.5 – 1.6)
Day 2	16.9 ± 25.7 (0.5 – 0.6)
Day 3	22.5 ± 28 (0.9 – 106.3)
Day 4	56.2 ± 73.2 (11.8 – 140.7)
Day 5	74.6 ± 83.4 (0.8 – 186.5)
IgM (x10^3 pg/mL)	2.6 ± 1.5 (2.0 – 7.3)
Day 1	2.3 ± 0.5 (1.9– 2.7)
Day 2	203.8 ± 531.3 (1.9– 1408)

Day 3	2649.4 ± 10913 (1.9 – 45000)
Day 4	2.6 ± 1.1 (1.9 – 3.8)
Day 5	3.1 ± 2.4 (1.9 – 6.8)
IgA (x10^3 pg/mL)	2207.6 ± 1067.6 (322.0 – 5021.0)
Day 1	2339 ± 302 (2125 – 2553)
Day 2	1944 ± 1311 (322 – 4025)
Day 3	2364 ± 777 (1460 – 4061)
Day 4	1394 ± 921 (450 – 2292)
Day 5	3936 ± 1534 (943 – 5021)
**Bacteria genus†**	
DNA Universal (x10^{-3} fg)	48.0 ± 180.0 (0.1 – 990.0)
Day 1	2.4 ± 1.7 (1.2 – 3.6)
Day 2	19.5 ± 29.4 (1.2 – 80.8)
Day 3	93.2 ± 254.4 (0.1 – 996.3)
Day 4	10.87 ± 6.2 (4 – 15.3)
Day 5	2.6 ± 3.4 (0.1 – 7.6)
DNA *Bifidobacterium* (x10^{-3} fg)	0.4 ± 0.8 (0.0 – 4.3)
Day 1	2.6 ± 3.4 (0.1 – 7.6)
Day 2	0.2 ± 0.1 (0.02 – 0.5)
Day 3	0.3 ± 0.2 (0.1 – 1)
Day 4	0.32 ± 0.27 (0.1 – 0.6)
Day 5	0.1 ± 0.01 (0.1 – 0.4)
DNA *Lactobacillus* (x10^{-3} fg)	0.9 ± 1.8 (0.0 – 9.3)
Day 1	0.1 ± 0.07 (0.1 – 0.2)
Day 2	0.4 ± 0.3 (0.003 – 1.2)

Day 3	0.9 ± 1.1 (0.000007 – 4.1)
Day 4	3.1 ± 5.3 (0.003 – 9.3)
Day 5	7.8 ± 0.9 (0.003 – 9.3)
DNA *Enterococcus* (x10^{-3} fg)	2.3 ± 1.0 (0.0 –44.0)
Day 1	0.0001 ± 0.00008 (0.00003 – 0.0001)
Day 2	4.4 ± 13.3 (0.000002 – 44.1)
Day 3	3.7 ± 12.1 (0.000003 – 43.1)
Day 4	0.0007 ± 0.0006 (0.0002 – 0.0001)
Day 5	0.00003 ± 0.00002 (0.000006 – 0.00005)
DNA *Streptococcus* (x10^{-3} fg)	31.0 ± 76.0 (0.0 – 320.0)
Day 1	15.7 ± 19 (2.3 – 29.2)
Day 2	124.1 ± 154.5 (1 – 323)
Day 3	11.7 ± 15.4 (0.0004 – 44.5)
Day 4	25 ± 17 (0.8 –25)
Day 5	1.1 ± 1.2 (0.3 –2.6)
DNA *Staphylococcus* (x10^{-3} fg)	30.0 ± 110.0 (0.0 – 480.0)
Day 1	18.4 ± 24.2 (1.2 – 35.6)
Day 2	122.5 ± 243.5 (0.3 – 487.9)
Day 3	6.5 ± 12.7 (0.01 – 39.5)
Day 4	0.02 ± 0.035 (0.05 – 0.0002)
Day 5	0.09 ± 0.16 (0.0002 – 0.29)
